# Self-testing for SARS-CoV-2 in São Paulo, Brazil: results of a population-based values and attitudes survey

**DOI:** 10.1186/s12879-022-07706-7

**Published:** 2022-09-02

**Authors:** Guillermo Z. Martínez-Pérez, Sonjelle Shilton, Maíra Saruê, Hilton Cesario, Abhik Banerji, Deepshikha Batheja, João Paulo Cunha, Rachel Baptista, Janine Schirmer, Eleva Ivanova Reipold, Alvaro Machado Dias

**Affiliations:** 1 FIND, The Global Alliance for Diagnostics, Geneva, Switzerland; 2 Instituto Locomotiva, São Paulo, Brazil; 3 Center for Disease Dynamics, Economics & Policy (CDDEP), New Delhi, India; 4 Department of Nursing, Federal University of São Paulo, São Paulo, Brazil; 5 Clinical Neuroscience Lab, Department of Psychiatry, Federal University of São Paulo, São Paulo, Brazil

**Keywords:** COVID-19, Home diagnostics, Brazil, SARS-CoV-2 testing, Self-testing, Survey

## Abstract

**Background:**

Brazil is among the countries in South America where the COVID-19 pandemic has hit the general population hardest. Self-testing for SARS-CoV-2 infection is one of the community-based strategies that could help asymptomatic individuals at-risk of COVID-19, as well as those living in areas that are difficult for health personnel to reach, to know their infectious status and contribute to impeding further transmission of the virus.

**Methods:**

A population-based survey was conducted in November 2021, to assess the acceptability of rapid SARS-CoV-2 antigen self-testing among the population of São Paulo. Survey respondents were approached at more than 400 different street-points that were randomly selected using a five-stage randomization process. A 35-item structured questionnaire was used. Dependent variables for our analyses were the likelihood to use and willingness to pay for self-testing, and the likelihood of taking preventive measures to prevent onward transmission of SARS-CoV-2 following a reactive self-test result. Bivariate and multivariate regression analyses were performed.

**Results:**

Overall, 417 respondents (44.12% female) participated; 19.66% had previously had COVID-19 disease. A minority (9.59%) felt at high-risk of COVID-19. The majority of both females and males (73.91% and 60.09%, respectively) were in favor of the idea of SARS-CoV-2 self-testing. Overall, if self-tests were available, almost half of the sample would be very likely (*n* = 54, 12.95%) or likely (*n* = 151, 36.21%) to use one if they felt they needed to. Upon receiving a positive self-test result, the majority of respondents would communicate it (88.49%), request facility-based post-test counseling (98.32%), self-isolate (97.60%), and warn their close contacts (96.64%).

**Conclusion:**

Rapid SARS-CoV-2 antigen self-testing could be an acceptable screening tool in São Paulo. The population would be empowered by having access to a technology that would allow them to test, even if asymptomatic, when traveling, or going to work or school. If there is a surge in the incidence of cases, self-testing could be a good approach for mass case detection by Brazil’s already overstretched Unified Health System.

## Introduction

Since the first case of Coronavirus Disease (COVID-19) caused by the novel Severe Acute Respiratory Syndrome Coronavirus 2 (SARS-CoV-2) was reported in Brazil in February 2020 [1], more than 32.8 million confirmed COVID-19 cases have been reported in the country to the beginning of July 2022 [2]. During the course of the pandemic, it is likely that many SARS-CoV-2-infected Brazilians may have gone undetected due, on one hand, to the absence of COVID-19 symptoms and, on the other hand, to the difficulty in accessing testing via the country’s Unified Health System (SUS, *Sistema Único de Saúde*) [3, 4]. In Brazil, a country where more than half of its population is aged less than 29 years [5], it is reasonable to assume that a considerable proportion of the population, comprising many young individuals who were asymptomatic carriers of SARS-CoV-2, may not even have suspected they could be transmitting the virus to other individuals [6].

In addition to mass vaccination, which is not progressing without hindrances in Brazil [7], other evidence-based screening strategies are needed to make efficient use of scarce resources for healthcare in this 8.5 million km^2^ territory, to identify cases that, albeit asymptomatic, do play a role in SARS-CoV-2 transmission. A range of community-based screening strategies has been proposed in other contexts, such as self-sampling among travelers [8], routine home screening of school pupils and staff [9], and point-of-care molecular testing at drive-through specimen collection sites [10]. These strategies have relied on the decentralization of SARS-CoV-2 testing. Rapid antigen testing for healthcare professional administration, self-sampling, and rapid antigen detection self-testing devices are among the technologies available to facilitate decentralization, with the aim of making mass screening for SARS-CoV-2 cases feasible in resource-constrained settings.

SARS-CoV-2 self-tests are rapid, lateral flow antigen detection assays; they have been regulated for public distribution or for over-the-counter sale in various countries, including Greece [11], Austria [12], the United States [13], and India [14]. These portable assays allow individuals to test themselves in private, at their own convenience, without the intervention of health personnel, and to learn their SARS-CoV-2 status in approximately 20 min [15, 16]. In essence, SARS-CoV-2 self-tests are very similar to professional rapid antigen tests, albeit the former must be marketed with packaging, test items, and user instructions tailored in such a way that any lay person can self-administer them safely [16–18].

Performance and usability studies of SARS-CoV-2 self-tests have demonstrated their accuracy [18, 19]. Earlier diagnosis, isolation, contact tracing, and access to treatment could be among the potential public health advantages of making SARS-CoV-2 self-testing available to the public [20]. However, acceptability studies are also necessary alongside performance studies, to try to gain an understanding of the public’s perceptions in relation to the value, utility, pertinence, and usability of self-tests. Self-testing for SARS-CoV-2 could be widely accepted among symptomatic and asymptomatic end-users in middle-income economies, with areas of the territory that are difficult for healthcare workers to reach. Brazil is not only a vast territory where the provision of medical coverage is challenging but also a society where niche groups of vulnerabilized populations, such as men who have sex with men and transgender persons have expressed the acceptability of self-testing to detect Human Immunodeficiency Virus (HIV) [21, 22] and Hepatitis C Virus (HCV) [23]. Limited access to conventional facility-based testing due to a lack of finances, remote locations, or the impossibility to forgo daily wages for many of those in the low-income generating sectors are common barriers to testing in Brazil; however, as self-testing for HIV [21, 22, 24] and HCV [23] has to some extent circumvented these barriers, they could also be circumvented by SARS-CoV-2 self-testing.

In January 2022, the Brazil Ministry of Health requested its National Health Surveillance Agency (*Anvisa*, as per its Portuguese acronym) to approve self-testing for the detection of SARS-CoV-2 in Brazil [25]. Two months later, the first Anvisa-approved self-test became available to the public in private pharmacies. To ensure SARS-CoV-2 self-testing becomes a game-changer, acceptability studies among end-users are necessary to inform regulatory and diagnostic practice. To tackle this knowledge gap, a population-based survey was conducted in São Paulo with the aim of assessing the population’s values in relation to rapid SARS-CoV-2 antigen self-testing (hereafter referred to as “self-testing”). Specific objectives of the survey were to understand factors that could predict: (i) the public’s likelihood to use self-testing, (ii) the public’s willingness to pay for a self-testing device, and (iii) the likelihood of an individual adhering to health authorities’ recommended actions following a positive self-test result.

## Methods

### Site and population

This population-based survey was conducted during November 2021 in the city of São Paulo, Brazil, a 1521 km^2^ metropolis inhabited by 12.4 million people and capital of a homonymous state. The survey population was São Paulo’s public. Eligibility criteria for all individuals were being aged 18 years or older, willing to provide consent, and free of COVID-19 symptoms at the time they were approached by the surveyors. It was estimated that 392 or more respondents were needed to have a confidence level of 95% that the real value (of willingness to use self-testing) was within ± 5% of the measured value.

### Sampling and recruitment

To limit any selection bias, a multi-staged sampling process was used. To begin with, São Paulo’s boundaries were defined using Google MyMaps®, and then the map was divided into 40 areas of similar width. These 40 areas excluded areas that were not possible to enter, such as those belonging to the military. Next, the 40 areas were randomly rearranged using RANDOM.ORG®. From this randomly rearranged list, the first 14 areas were selected as survey areas. Then, to determine the sequence that the surveyors would follow to visit the areas, the 14 areas were randomly rearranged, again using RANDOM.ORG®. Finally, in each area, 30 street-points were randomly selected.

The surveyors were tasked with recruiting one respondent per selected street-point. The survey was conducted over seven days. Upon arrival at each street-point, the surveyors stopped the first passer-by they saw and invited them to participate. If the person declined, the surveyors had to wait three minutes until they could stop a new passer-by, repeating this process until they found a person who was interested in participating.

### Data collection and analysis

Informed consent was obtained and data collection was performed either on-the-spot where privacy could be guaranteed or, if necessary, in a nearby site of the respondent’s choice.

A 35-point questionnaire was used; the questionnaire had been informed by previous FIND-led studies of communities’ values around self-testing for HCV [21]. It included items about respondents’ socio-demographics; previous experiences with conventional COVID-19 testing; knowledge of other self-tests; likelihood to use self-testing; willingness to pay for self-testing; barriers to using self-testing; and likely actions upon self-testing positive or negative for SARS-CoV-2 [26]. The questionnaire was designed in English, translated into Portuguese, and pre-piloted in São Paulo among staff in the premises of the survey implementing organization, Instituto Locomotiva. When the questionnaire was considered suitable, it was developed in KoBoToolbox®, re-tested, and deployed in the KoBoCollect® application.

The surveyors collected data from all respondents using their tablet-enabled KoBoCollect® application and submitted the responses immediately. No personal identifiers were collected. Submissions were monitored daily for data inaccuracies or incompleteness. Once the survey ended, all data were exported into an MS-Excel ® file for analysis.

Descriptive statistics and bivariate and multivariate analyses were run in STATA v.14®. The primary outcomes of the analysis were likelihood to use self-testing, willingness to pay for a self-testing device, and likelihood to comply with hygiene and prevention of transmission recommendations upon receipt of a reactive self-test result (i.e., report the result, self-isolate, identify contacts, wear a face mask). Significant associations were sought between the primary outcomes and the respondents’ characteristics, and other aspects of interest to inform future self-testing distribution programs, which were inclusive of but not limited to the perception of risk of COVID-19 disease; awareness of other self-testing devices; and previous experience with conventional SARS-CoV-2 testing. Variables that were significantly associated with the primary outcomes at a *P*-value of < 0.05 were entered into a multivariate regression model. A logistic regression model was used to identify potential predictors of likelihood to use and willingness to pay for self-testing. Ordinary Least Squares (OLS) regression was used to identify predictors of compliance with hygiene and prevention of transmission recommendations upon receipt of a reactive self-test result.

### Ethics

All respondents gave their informed consent to participate. Respondents signed two copies of the consent document and kept one signed copy. The survey protocol received ethical clearance from the Research Ethics Committee of the Federal University of São Paulo (UNIFESP-CEP).

## Results

### Participants’ characteristics and experiences with COVID-19

In total, 184 (44.12%) females (mean age 40.1 years (Standard Deviation (SD) 15.53) and 233 (55.87%) males (mean age 45.2 years (SD 15.02) participated (Table 1). Most female participants were in the 18–35 age group (*n* = 85; 46.20%), while most male participants were in the 36–55 age group (*n* = 97; 41.63%). All respondents self-identified as Brazilian nationals, with white (*branco* in Portuguese) (*n* = 169; 40.63%), brown (*pardo*) (*n* = 153; 36.78%), and black (*preto*) (*n* = 78; 18.75%) being the most self-reported ethno-racial identities.

**Table 1:** Respondents’ characteristics and experiences with COVID-19

	Female *n* = 184 (44.12%)	Male *n* = 233 (55.87%)	*P*-value	Total *n* = 417 (100.00%)
Mean age (SD), years^a^	40.125 (SD 15.553)	45.262 (SD 15.024)	< 0.001	42.995 (SD 15.454)
Age range (years)^a^			< 0.001	
18–35	85 (46.20)	69 (29.61)		154 (36.93)
3 6–55	65 (35.33)	97 (41.63)		162 (38.85)
≥ 56	34 (18.48)	67 (28.76)		101 (24.22)
Ethno-racial identity^a^^, b^			0.552	
White (*Branco*)	74 (17.68)	95 (22.83)		169 (40.63)
Black (*Preto*)	30 (7.21)	48 (11.53)		78 (18.75)
Brown (*Pardo*)	69 (16.58)	84 (20.19)		153 (36.78)
Indigenous (*Indígena*)	10 (2.40)	1 (0.24)		11 (2.64)
Asian (*Amarelo*)	0 (0.00)	1 (0.24)		1 (0.24)
Education^a^			0.225	
None	19 (10.33)	16 (6.87)		35 (8.39)
Primary	42 (22.83)	79 (33.91)		121 (29.02)
Secondary	78 (42.39)	109 (46.78)		187 (44.84)
College/vocational	14 (7.61)	8 (3.43)		22 (5.28)
Degree	25 (13.59)	15 (6.44)		40 (9.59)
Postgraduate	4 (2.17)	0 (0.00)		4 (0.96)
PhD	0 (0.00)	1 (0.43)		1 (0.24)
Other	2 (1.09)	5 (2.15)		7 (1.68)
Employment status^a^			< 0.001	
Unemployed	40 (21.74)	26 (11.16)		66 (15.83)
Student	5 (2.72)	4 (1.72)		9 (2.16)
Employed, part-time	6 (3.26)	3 (1.29)		9 (2.16)
Employed, full-time	67 (36.41)	60 (25.75)		127 (30.46)
Self-employed	32 (25.00)	117 (50.22)		163 (39.09)
Retired, on a pension	20 (10.87)	23 (9.87)		43 (10.31)
Feeling at-risk of COVID-19^a^			0.743	
No risk	11 (5.98)	15 (6.44)		26 (6.24)
Low risk	40 (21.74)	59 (25.32)		99 (23.74)
Mild risk	62 (33.70)	72 (30.90)		134 (32.13)
Moderate risk	49 (26.63)	69 (29.61)		118 (28.30)
High risk	22 (11.96)	18 (7.73)		40 (9.59)
Having had COVID-19^a^			0.011	
Yes, confirmed by test	39 (21.20)	28 (12.02)		67 (16.07)
Yes, confirmed by a healthcare worker	5 (2.72)	10 (4.29)		15 (3.60)
For those tested	57 (13.66)	50 (11.99)	0.016	107 (25.65)
Months ago (mean, SD)	9.19 (9)	77.38 (8)		8.34 (8)
Paid for the test	18 (20.22)	21 (14.48)	0.735	39 (16.67)
Amount paid (mean USD, SD)	3.93 USD (SD 8.25)	2.91 USD (SD 7.56)		3.3 USD (SD7.83)

^a^Percentages took into consideration missing values for each variable, i.e., they were not calculated based on the total sample of 417 respondents

^b^In brackets, ethno-racial identity terms used in Brazilian Portuguese

Regarding education, the majority (*n* = 187; 44.84%) had completed secondary education (Table 1). Completion of primary studies only was higher in males (*n* = 79; 33.91%) than females (*n* = 42; 22.83%), whereas completion of university undergraduate and postgraduate degrees was higher among females (*n* = 29; 15.76%) than males (*n* = 16; 6.83%). Regarding employment, unemployment was more frequent among females (*n* = 40; 21.74%) than males (*n* = 26; 11.16%); employment by a third party was more commonly reported among females (*n* = 73; 39.67%) than males (*n* = 63; 27.04%); and self-employment was more common among males (*n* = 117; 40.22%) than females (*n* = 32; 25.00%).

Regarding perception of risk of COVID-19, 134 (32.13%) and 118 (28.30%) respondents felt they were at mild- and moderate-risk of COVID-19, respectively (Table 1). In comparison, a minority (*n* = 40; 9.59%) felt they were at high-risk of COVID-19. Almost one in four respondents (*n* = 92; 22.06%) considered that they were sharing a household with elders who were at risk of severe COVID-19 disease, although 55.64% (*n* = 232) of respondents, perceived that they were not living with any individual at increased risk of COVID-19. There were 82 (19.66%) respondents who had COVID-19 disease; of these, 67 (16.07%) had their SARS-CoV-2 infection confirmed by a test.

Of the sample, 57 (13.66%) females and 50 (11.99%) males reported having ever received a SARS-CoV-2 test (Table 1). Among these respondents, their most recent test was an average of 8.34 (SD 8) months ago. Of these 107 (25.65%) respondents, 18 (4.31%) females and 21 (5.03%) males paid for their most recent test, with a mean cost of 3.93 United States Dollars (USD) (SD 8.25) and 2.91 USD (SD 7.56), respectively.

Sex at birth was a variable that could statistically explain differences in responses to age, employment and previous access to SARS-CoV-2 testing (all *P* < 0.05) but not to education, self-expressed ethno-racial identity, risk perception or paying for their most recent test (Table 1).

### Acceptability of self-testing

When asked about their awareness of other self-testing devices, 149 (80.98%) females and 178 (76.39%) males knew about self-tests for pregnancy, while 40 (21.74%) females and 31 (13.30%) males knew about SARS-CoV-2 self-testing (Table 2). Overall, 139 (33.33%) respondents agreed with the idea or concept of people being able to self-test at home for any infectious disease. Notably, the rate of agreement doubled when the respondents were asked specifically about their agreement with self-testing for SARS-CoV-2, with 73.91% (*n* = 136) of females and 60.09% (*n* = 140) of males expressing their agreement. Additionally, if provided free of charge and recommended by health authorities, 44.57% (*n* = 82) of females and 39.48% (*n* = 92) of males would be willing to self-test on a weekly basis. Sex at birth was a respondent characteristic that could statistically explain differences in responses to agreement with the concept of SARS-CoV-2 self-testing (*P* = 0.003).

**Table 2:** Acceptability of self-testing for SARS-CoV-2

	Female *n* = 184 (44.12%)	Male *n* = 233 (55.87%)	*P*-value	Total *n* = 417 (100.00%)
Awareness of self-testing devices^a^			0.0229	
SARS-CoV-2	40 (21.74)	31 (13.30)		71 (17.03)
HIV	2 (1.09)	0 (0.00)		2 (0.48)
HCV	1 (0.54)	0 (0.00)		1 (0.24)
Hypertension	14 (7.61)	20 (8.58)		34 (8.15)
Pregnancy	48 (26.09)	68 (29.18)		116 (27.82)
Diabetes/glycaemia	149 (80.98)	178 (76.39)		327 (78.42)
Agreement with the concept of SARS-CoV-2 self-test^a^	136 (73.91)	140 (60.09)	0.003	276 (66.19)
Willingness to pay	139 (75.54)	150 (64.38)	0.014	289 (69.30)
Amount, USD (mean, SD)	5.64 USD (SD 4.35)	5.63 USD (SD 5.06)	0.99	5.64 USD (SD 4.73)
Likelihood of using a self-test^a^			0.596	
Very unlikely	67 (36.41)	63 (27.04)		130 (31.18)
Unlikely	14 (7.61)	37 (15.88)		51 (12.23)
Neutral	11 (5.98)	20 (8.58)		31 (7.43)
Likely	67 (36.41)	84(36.05)		151 (36.21)
Very likely	25 (13.59)	29 (12.45)		54 (12.95)

^a^Percentages took into consideration missing values for each variable, i.e., they were not calculated based on the total sample of 417 respondents

**Fig. 1:**
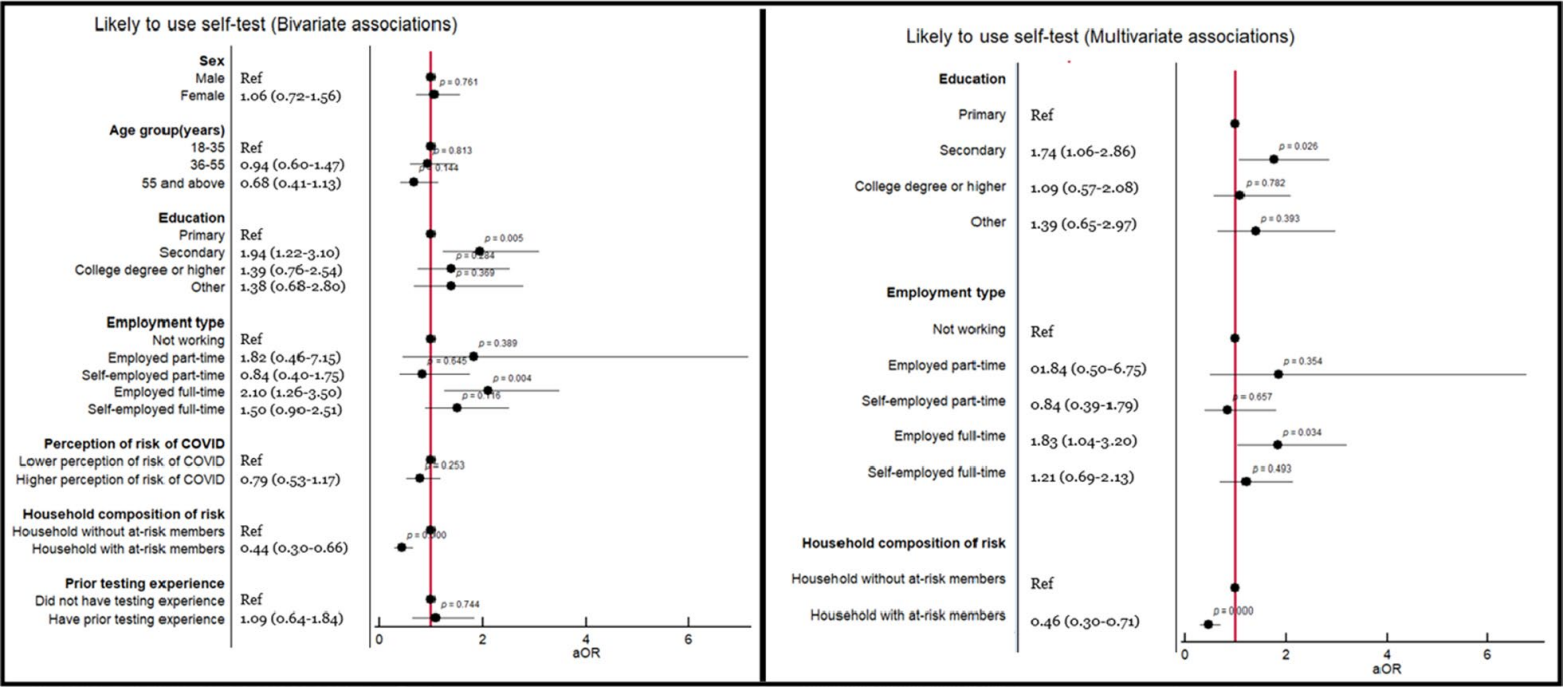
Bivariate and multivariate logistic regression analyses depicting the association between independent variables and likelihood to use a self-test kit

Likelihood to use SARS-CoV-2 self-testing when needed resulted in an overall rating of 2.832/5 (SD 1.557) for females and 2.91/5 (1.449) for males. Overall, if self-tests were available in Brazil, almost half of the sample would be very likely (*n* = 54, 12.95%) or likely (*n* = 151, 36.21%) to use them. The multivariate model (Fig. 1) showed that those having secondary education (Adjusted Odds Ratio (AOR): 1.75, 95% Confidence Interval (95% CI): 1.07–2.86, *P* = 0.026) or working full-time for an employer (AOR: 1.83, 95% CI 1.046–3.20, *P* = 0.034) had comparatively higher odds of using self-testing; while those who lived in a household with individuals at increased risk of severe COVID-19 disease (AOR: 0.467, 95% CI 0.306–0.71, *P* < 0.001) had comparatively lower odds of using self-testing.

**Fig. 2:**
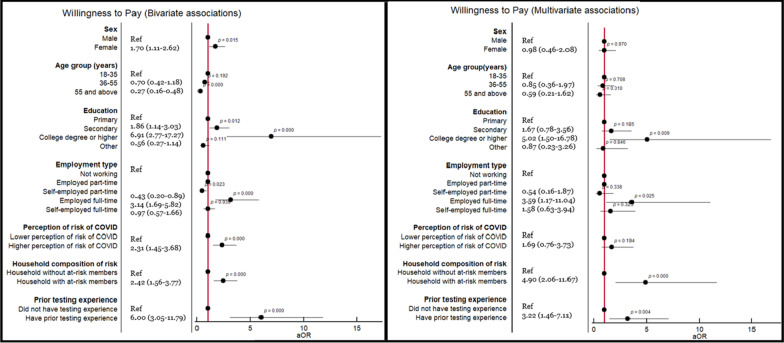
Bivariate and multivariate logistic regression analyses depicting the association between independent variables and willingness to pay for COVID-19 self-testing kits

Overall, 289 (69.3%) respondents expressed that they would be willing to pay for self-testing, with a mean of 5.64 USD (SD 4.73) (Table 2). Those respondents aged ≥ 56 years (AOR: 0.482, 95% CI 0.306–0.71, *P* < 0.001) and those self-employed part-time (AOR: 0.394, 95% CI 0.17–0.91, *P* = 0.03) had comparatively lower odds of paying for self-testing (Fig. 2). The respondents with a college degree or higher (AOR: 4.2, 95% CI 1.6–10.94, *P* = 0.003), working full-time (AOR: 2.1, 95% CI 1.04–4.32, *P* = 0.038), who perceived themselves to be at high-risk of COVID-19 (AOR: 1.88, 95% CI 1.12–3.17, P = 0.017), and who lived in a household with individuals at increased risk of severe COVID-19 disease (AOR 2.58, 95% CI 1.52–4.36, *P* < 0.001) had comparatively higher odds of paying for self-testing kits (Fig. 2).

### Actions upon self-testing for SARS-CoV-2

Most respondents stated that, should they perform a self-test and its result were positive, they would go in person to a health facility to request post-test counseling (*n* = 410, 98.32%), self-isolate (*n* = 407, 97.60%), warn their close contacts (*n* = 403, 96.64%), and communicate the result (*n* = 369, 88.49%) (Table 3). One in ten (*n* = 42; 10.07%) respondents expressed that they would not report a positive result.

**Table 3:** Actions taken following a SARS-CoV-2 self-test

	Female *n* = 184 (44.12%)	Male *n* = 233 (55.87%)	*P*-value	Total *n* = 417 (100.00%)
Practice following receipt of a positive self-test result				
Communicate the result to a clinic, hospital and/or COVID hotline^a^	164 (89.13)	205 (87.98)	0.71	369 (88.49)
Go in person to a clinic or hospital to seek post-testing counseling from a healthcare worker^a^	180 (97.83)	230 (98.71)	0.485	410 (98.32)
Self-isolate^a^	180 (97.83)	227 (97.42)	0.79	407 (97.60)
Identify and warn close contacts^a^	178 (96.74)	225 (96.57)	0.92	403 (96.64)
Inform their employer^a^	171 (95.00)	379 (79.45)	0.093	278 (92.98)
Practice following receipt of a negative self-test for a person with symptoms and exposed to a COVID-19 case				
Stop self-isolation^a^	164 (89.13)	209 (89.70)	0.76	373 (89.45)
Stop wearing a face mask^a^	22 (11.96)	21 (9.01)	0.21	43 (10.31)
Stop social distancing^a^	58 (31.52)	54 (23.18)	0.16	112 (26.86)

^a^Percentages took into consideration missing values for each variable, i.e., they were not calculated based on the total sample of 417 respondents

There was a slight difference in responses regarding communication of a positive result to an employer, with 95.00% (*n* = 171) of females with an income-generating activity and 79.45% (*n* = 379) of males with an income-generating activity reporting that they would report a positive result to their employer (Table 3).

In the event that a respondent knew that they had been exposed to a person who had COVID-19 and that the respondent had COVID-19-related symptoms but received a negative self-test result, the majority of respondents (*n* = 373, 89,45%) would stop self-isolating (Table 3). However, in this hypothetical scenario, a minority would not stop social distancing (just *n* = 112, 26.86% would) and would not stop wearing face masks (just *n* = 43, 10.31% would).

OLS regression found no significant association with any independent variable.

## Discussion

Our survey shows that there is potential for the acceptability of SARS-CoV-2 self-testing in Brazil. In our inquiry, acceptability was conceptualized as a composite of the public’s values toward self-testing, including agreement with the concept of self-testing (73.91% and 60.09% of female and male respondents were in agreement, respectively); willingness to pay for self-testing (if available at an average price of 5.64 USD (SD 4.73) for the 69% of respondents who would pay for a self-test device); willingness to serially self-test (44.57% of females and 39.48% of males expressed willingness to perform weekly self-tests); and likelihood to use self-testing (12.95% and 36.21% of respondents were “very likely” or “likely”, respectively, to use a self-test). Although satisfactory, the rates of likelihood to use a self-test were not as high as those found in Indonesia [26], Nigeria [27], or Kenya [28], other countries where surveys of people’s values and attitudes towards self-testing were conducted in 2021 using the same methodology we used in São Paulo.

People’s attitudes toward the acceptability of self-diagnostics are context-dependent, and can be mediated by cost, design, accuracy, accessibility, and health authorities’ endorsement of self-testing for infectious diseases, among other factors. Our survey was designed to assess what the predictors of acceptability might be in a variety of countries [25]. As per our regression analyses, individuals with a secondary education or who are working full-time for an employer might have higher odds of being likely to using a self-test when needed, while those living in a household with people at increased risk of severe COVID-19 disease might have lower odds of being likely to using a self-test. The regression analyses also suggested that individuals aged ≥ 56 years and those self-employed part-time may have lower odds of paying for a self-test device, while individuals with higher education, those who are working full-time, those who perceive themselves to be at high-risk of COVID-19, and those living in a household with individuals at increased risk of severe COVID-19 disease might have higher odds of paying for a self-test device. The predictors we detected might be helpful for those planning community- and primary healthcare-based testing services to map the profiles of the population groups who might be more attracted to using (or not) self-testing and, thereafter, to decide who should be targeted via the promotion of self-testing in São Paulo. Nevertheless, we also warrant caution in considering only the predictors detected by our analyses in future self-testing promotion planning. As suggested by HIV self-testing experiences [20, 21, 23, 29], the more that SARS-CoV-2 self-testing programs are diverse, inclusive, civil society-endorsed, and decentralized, the more likely it will be that such programs will efficiently meet the needs of different sectors of the public. To be responsive to these needs, programs must take full consideration of the intersectionality of populations’ barriers to accessing testing and care with their personal cultural, clinical, and socio-economic characteristics.

The drivers for the acceptability of self-testing that our survey explored are dependent on the studied population’s historical, sociocultural, and epidemiological context. Factors that may mediate the general public’s acceptability of self-testing in Brazil, such as access to facility-based SARS-CoV-2 testing and vaccination programs, and the epidemiological evolution of the pandemic, have undergone frequent changes since February 2020. These are factors that, for different persons and in different moments and geographies, might act as deterrents or as drivers of the acceptability and uptake of self-testing. The rapidly changing epidemiological scenario and public health authorities’ responses since the first SARS-CoV-2 infection was reported in Brazil are among the reasons why for our survey methodological approach we considered respondents’ characteristics, and not their context, as predictors of acceptability.

It must be noted that, regarding our respondents’ context, as of January 2022, after our survey had already ended, Brazil’s regulatory authorities (i.e., *Anvisa*) were beginning the process of accepting companies’ requests for approval for distribution of their SARS-CoV-2 self-tests [24, 30, 31]. In mid-February 2022, the *Anvisa* approved the first device (i.e., the CPMH® COVID-19 antigen self-test) for distribution [32]. In this context, depending on how self-testing is introduced and explained to the Brazilian general public and healthcare workforce, individuals’ values and preferences for access and usage of this case detection approach may be impacted. Indeed, the more user-friendly SUS health facilities are to self-testers, the more likely it will be that self-testers react favorably to a reactive result. The more transparent the government is in providing evidence that self-testing can decrease the incidence of COVID-19-related morbidity and mortalitu, the more likely it is that the public, and especially daily laborers, education center attendees, and those interested in traveling or in attending social gatherings, might want to self-test more frequently.

Of the self-testing acceptability studies that have been conducted, our survey findings are aligned with the results of studies conducted in Germany [9], Indonesia [26], Nigeria [27], Kenya [28], the United Kingdom [33], and Greece [34, 35] and Cyprus [34], where the study populations also manifested a willingness to use self-testing. Of these studies, only the inquiries in Indonesia [26], Kenya [28], Greece [34, 35] and Cyprus [34] targeted the general public. Comparing our survey with that of Goggolidou et al. [34] and Mouliou et at. [35], it should be noted that different contextual factors might have mediated the respondents’ favorable opinions exhibited toward self-testing in each study. In Greece, the health authorities had approved self-testing, distributed self-tests free of charge, and had made educational materials for end-users available via a government website [11]. These efforts could have promoted favorable public opinion toward self-testing in Greece. Nevertheless, it is worth noting that the survey carried out by Mouliou et al. [35] in mainland Greek reported that almost half of the total sample (*n* = 614) considered self-testing *‘dangerous’*, and that only one in five respondents declared that they would buy a self-test. And, to our knowledge, this survey in Greece [35] is the only comparing attitudes towards self-testing in populations with both exposure to and experience of self-testing and facility-based testing for SARS-CoV-2.

It can be hypothesized that the public response to the Brazilian government’s behavior with regards to the COVID-19 pandemic in Brazil might have influenced people’s willingness to self-test, especially as our data collection was conducted prior to the surge of the Omicron variant of SARS-CoV-2, which dramatically increased the local (as much as the global) demand for rapid antigen-detection tests for SARS-CoV-2. In Brazil, the government’s challenges to providing mass screening and testing have been acknowledged [3, 4]. In mid-January 2022, a phone survey revealed that—in the midst of the Omicron variant wave—more than 8.1 million Brazilians had tried and failed to obtain a SARS-CoV-2 test [36]. These challenges to accessing testing have driven many Brazilians to resort to private healthcare. In a previous study of self-testing for HCV, informants reported that they would prefer to either self-test or go to a private practitioner for HCV testing rather than to go to an SUS facility [23]. Similarly, now that *Anvisa* is receiving requests from manufacturers for the registration of self-tests [32], many people may opt to purchase a SARS-CoV-2 self-test via a private provider rather than trying to access facility-based testing. Although accessing self-testing in the private healthcare sector may alleviate the burden in overstretched SUS facilities, attention needs be paid to private facilities’ capacities to inform their clients on the risk of false results when SARS-CoV-2 incidence rates drop in Brazil [37]. The individuals’ clinical status and their vaccination and exposure history to SARS-CoV-2, or the prevalence of SARS-CoV-2 infections in the community at the time of self-testing are other factors that can lead to false results and that must be considered in self-testing delivery models [37].

Future studies may provide a more thorough indication of what the reasons might be for the acceptability of self-testing in contexts where these devices have not previously been widely deployed. Future studies will also have to discern what attitudes are triggered by the intrinsic advantages of self-testing, and which attitudes are triggered by health system-related failures to cater for individuals at risk of SARS-CoV-2 acquisition and of severe COVID-19 disease. While our survey findings are optimistic (i.e., 88.49%, 97.60%, and 96.64% of respondents would communicate their result, self-isolate, and warn their contacts, respectively), in actuality, post-self-testing behaviors might be different if no social, labor or family support is provided. If social safety nets are not provided, self-isolation and reporting of a SARS-CoV-2 infection might be neither feasible nor desirable for affected people. As other self-testing studies in Indonesia and Nigeria have suggested, self-isolation might only be guaranteed if there are provisions in place to ensure that those who use a self-test do not lose their job or social position [26, 27].

Our survey findings have other implications for practice. Ideally, self-tests should cost less than 5.64 USD, to enable people to afford them. Further education on the risk of false-negatives must be provided, as 89.45% of our respondents expressed that they would stop self-isolating if they self-tested negative, even if they were symptomatic and had been in contact with a case. It is possible that an emphasis on frequent testing might be needed, to counterbalance the effects of the likely lower sensitivity of some SARS-CoV-2 self-testing devices (despite the *Anvisa* requirement that self-tests for distribution in Brazil must have at least 80% sensitivity and 97% specificity [30]), so that individuals who suspect they might have SARS-CoV-2 repeat a self-test every 24 h and monitor their symptoms before deciding to stop self-isolating. While self-testing may have an added value to identify asymptomatic SARS-CoV-2 carriers—and, while self-testing programs may be useful to educate the public on the possibility to transmit the virus to other persons even if the carriers are asymptomatic-, culturally-grounded and less value-laden communication materials will be necessary to sensitize self-testing users on the implications of being, and sharing spaces with, an asymptomatic carrier.

Considering our findings, it could be argued that facilitating access to self-testing may be a useful case detection approach to halting or slowing the transmission of SARS-CoV-2. Self-testing has the potential to reduce the burden on SUS facilities, which should be attending to those who are most seriously ill. It also has the potential to be scaled-up in educational, religious or working environments, where large numbers of individuals regularly congregate. Self-testing could also be useful in the hands of civil society-based grassroots organizations that can promote community-based testing for SARS-CoV-2 in poverty-stricken *favelas* or in areas where indigenous populations are in urgent need of improved access to testing.

It should be noted that this survey had some limitations. First, the findings might be representative of the inhabitants of São Paulo city but not representative of people who live in rural areas of the state. The intention was to conduct the study throughout the entire state, but for logistical reasons and because of restrictions on social movement due to COVID-19, this was not possible. Also, it must be noted that to avoid security incidents within São Paulo city, recruitment in some *favelas* took place in the areas’ main avenues. It is not possible to know whether the results may have varied slightly if the interiors of these neighborhoods had not been avoided. Despite these impediments, we managed to recruit a diverse sample, with a broad representation of self-expressed ethno-racial identities, education levels and employment statuses.

## Conclusion

We carried out a survey on values and attitudes towards SARS-CoV-2 self-testing in São Paulo. Our findings suggest that the general public in São Paulo favors the idea of SARS-CoV-2 self-testing; would be likely to use self-test devices if available and affordable; and that, following a reactive self-test result, would request post-test counseling, self-isolate, and warn their contacts of their SARS-CoV-2 infection. Thus, SARS-CoV-2 self-testing would be an acceptable solution in São Paulo and possibly most of Brazil for those individuals who suspect that they may be infected with SARS-CoV-2 or are a close contact of a person with the infection, who want to travel, or who want to ensure the safety of themselves and their peers if they go to school or work. To increase the opportunities for the safe and effective uptake of self-testing, health authorities should regulate to ensure that self-testing is a viable complement to facility-based, conventional SARS-CoV-2 testing for symptomatic individuals and their contacts.

## Data Availability

The datasets used and/or analyzed during the current study are available from the corresponding author on reasonable request.
